# High and low dose radiotherapy combined with ICIs for MSS colorectal cancer patients with liver metastases: a phase I study (HaRyPOT)

**DOI:** 10.3389/fonc.2025.1503517

**Published:** 2025-02-06

**Authors:** Yuxuan Ding, Yong Feng, Yangfan Ye, Jiayi Shen, Chang Guo, Xia He, Liangjun Zhu, Lijun Wang

**Affiliations:** ^1^ Department of Radiotherapy, The Affiliated Cancer Hospital of Nanjing Medical University, Jiangsu Cancer Hospital, Jiangsu Institute of Cancer Research, Nanjing, China; ^2^ Department of Neurosurgery, The First Affiliated Hospital of Nanjing Medical University, Nanjing, China

**Keywords:** colorectal cancer, microsatellite-stable (MSS), low dose radiotherapy, tumor environment, immune checkpoint inhibitors

## Abstract

**Introduction:**

Microsatellite stable (MSS) colorectal cancer with liver metastases (CLRM) responds poorly to immunotherapy, and various approaches to break immune tolerance have been tried. Radiotherapy in combination with immune checkpoint inhibitors is one of promising therapies, and the choice of radiotherapy and immunotherapy modalities is also a hot issue.

**Methods:**

Here, we report on a Phase I trial treating nine patients with MSS CLRM using a combination of high and low dose radiotherapy and immune checkpoint inhibitors (ICIs).

**Results:**

The primary endpoint of the trial was the safety and tolerability of this combination treatment modality. Secondary endpoints included the objective response rate (ORR), progression-free survival (PFS) and overall survival (OS). The study results showed that at three dose levels—single doses of 6Gy (n=3), 8Gy (n=3), and 10Gy (n=3)—the most common treatment-related adverse events (TRAEs) were nausea, vomiting, fatigue, and abnormal liver function. At the first condition assessment, four patients were observed to have stable disease (SD) and one patient achieved partial response (PR). In exploratory endpoint analyses, tissue biopsies and paired hematologic samples from patients showed M2 macrophage reduction. Plasma cytokines IL-10, IL-17, and INF-α increased after treatment with both drugs.

**Discussion:**

In summary, this is the first clinical trial demonstrating the safety and immunogenic activity of combined high and low dose radiotherapy with ICIs in MSS colorectal cancer liver metastases (CRLMs). The combination therapy stimulated the immune response and altered the tumour microenvironment, warranting further exploration in the future.

## Background

The incidence and mortality rates of colorectal cancer are among the highest of all malignant tumors (1, 2). Immunotherapy is ineffective for the majority of patients with microsatellite-stable (MSS) colorectal cancer (CRC) due to the tumor’s low immunogenicity and suppressive tumor microenvironment (TME) (3, 4). This results in an even slimmer chance of treatment for this group of patients. Additionally, more than half of the patients eventually develop liver metastases (5, 6). In a study by Fakih et al., the combination of nivolumab and regorafenib for advanced colorectal cancer showed an overall response rate (ORR) of 21.7% in patients without liver metastases, compared to 0% in those with liver metastases (7). How to improve the response rate to immunotherapy in MSS CRLM patients, researchers have tried various methods to enhance the immunostimulatory effects of immune checkpoint inhibitors and to break immunosuppression. The combination of radiotherapy and immune checkpoint inhibitors is one of the directions explored.

Preclinical studies have shown that high doses of radiotherapy cause immunogenic death of tumor cells, releasing large amounts of tumor-specific antigens, and attracting downstream cytokines and T cells to the TME (8, 9). Combining high-dose radiotherapy with immune checkpoint inhibitors has yielded significant results in non-small cell carcinoma (NSCLC), with patients’ progression free survival (PFS) and 3-year overall survival (OS) greatly improved (10). Meanwhile, low dose radiotherapy (LDRT) also plays a unique role, which produce a large amount of chemokines, facilitating the recruitment of effector T cells into the TME (11, 12). In 2022, a study showed that low-dose whole-abdominal radiotherapy (LD-WART) combined with immune checkpoint blockade (ICB) resulted in tumor responses in 83.5% of immune-resistant ovarian cancer mice, with a cure rate of 15% (13). A subsequent clinical trial employing a similar combination therapy in 8 patients with “immune desert” tumors showed an ORR of 12.5% and a grade 3 adverse event rate of only 25%. High dose radiotherapy releases tumor antigens and low dose radiotherapy modulates the tumor microenvironment. The combination of the two appears to greatly stimulate the immune response and amplify the effects of immune checkpoint inhibitors. Indeed there have been trials on melanoma that have confirmed the effectiveness of triple therapy, the combination of SBRT + LDRT + CTLA-4 inhibitor improved distant effects and survival, and mice developed prolonged immune memory after treatment (14).

Therefore, we conducted a phase I study combining high and low dose radiotherapy with ICIs to treat patients with MSS CLRM. The aim of the study is to investigate the anti-tumor safety, tolerability and activity of this triple therapy on patients with MSS CLM, and to preliminarily explore its impact on the immune environment.

## Methods

### Study design

This is a single-center, single-arm, Phase I study combining high and low dose radiotherapy with ICIs in the treatment of MSS CRLM. The study was approved by the Ethics Committee of Jiangsu Cancer Hospital (approval ID: 2023-046) and was designed and conducted in accordance with the Declaration of Helsinki and ethical guidelines for clinical research. All participants provided written informed consent. The study is registered on ClinicalTrials.gov (Identifier: NCT06045286).

### Patient enrollment

Eligible patients were those aged ≥18 years with pathologically confirmed MSS CRLM, deemed currently unresectable by multidisciplinary treatment (MDT). All patients must have progressed on at least two lines of systemic therapy. Patients needed to have sufficient organ function to tolerate radiotherapy and have other assessable lesions aside from the high-dose irradiation target in the liver. The complete eligibility criteria are defined in the **Supplementary Materials**.

### Treatment protocol

The researchers selected 1-4 liver lesions in patients for high dose radiotherapy. While delivering high dose radiotherapy, low-dose radiotherapy was administered to other irradiable liver lesions. Two weeks after completing radiotherapy, patients received immune checkpoint inhibitor treatment. Paired hematologic and liver tumor tissue biopsy samples were collected before and after treatment. The study protocol is provided in **Supplementary Materials**.

#### 1. Radiotherapy

High-dose irradiation was administered using stereotactic body radiotherapy (SBRT), and low-dose irradiation using Volumetric Modulated Arc Therapy (VMAT). The treatment process included CT simulation positioning, plan verification, and execution. During CT simulation positioning, patients were in the supine position, fixed with a thoracoabdominal vacuum bag, with both upper limbs raised and abducted, and hands interlocked above the head. The scanning range extended 3-5 cm above the upper liver border and 3-5 cm below the lower liver border, with a slice thickness of 2.5 mm. Target delineation utilized CT and fused MRI/PET-CT images. The high-dose gross tumor volume (HD-GTV) consisted of 1-4 tumor lesions, with single doses of 6-10 Gy, administered over 3-5 consecutive sessions. The maximum diameter of tumors irradiated at high doses is 5 cm. The low-dose gross tumor volume (LD-GTV) received single doses of 1.2 Gy, administered over 3-5 consecutive sessions. The lesion to be irradiated is selected on a patient-by-patient basis by at least 2 radiotherapy specialists and the extent of the target area is outlined, and the treatment plan calls for 100% coverage of the GTV with the prescribed dose. Organ-at-risk constraints referred to the normal tissue tolerance dose (QANTAC) table.

#### 2. Immune checkpoint inhibitor

ICIs were administered two weeks after the end of radiotherapy. The immune checkpoint inhibitor selected was zimberelimab, administered via intravenous infusion. The dosage for intravenous infusion was 240 mg every 3 weeks, continued until disease progression or the development of intolerable toxicity.

### Clinical endpoints

The primary objective of the study was to evaluate the safety of combining high and low dose radiotherapy with ICIs in patients with MSS CRLM. The secondary objective were objective response rate (ORR, CR+PR), median progression-free survival (PFS), and overall survival (OS). The exploratory objectives were to characterize immunological responses. PFS was defined as the time between enrolment and disease progression or death. OS was defined as the time from enrolment to death from any cause.

#### 1. Radiographic response evaluation

Patients who received two cycles of zimberelimab injections after radiotherapy could undergo efficacy evaluation. Disease status was assessed every 8 weeks using contrast-enhanced CT scans or magnetic-resolution imaging (MRI). Radiological responses and disease progression were evaluated by researchers according to the Response Evaluation Criteria in Solid Tumors (RECIST) v1.1.

#### 2. Safety and tolerability

Safety analysis included all patients who received at least one dose of zimberelimab. Adverse events related to the treatment were descriptively reported according to the National Cancer Institute’s Common Terminology Criteria for Adverse Events (CTCAE) v5.0.

#### 3. Immuno-related analysis

Multiple analyses of immunomarkers were performed on formalin-fixed, paraffin-embedded fine-needle biopsies of paired samples. Sections were sequentially stained for Foxp3 (ab20034, abcam), CD4 (ZA0519, ZhongShan Golden Bridge, Beijing), CD8 (ZA0508, ZhongShan Golden Bridge, Beijing), CD68 (ZM-0060, ZhongShan Golden Bridge, Beijing), ab64088, abcam), and CD163 (ab189915, abcam). All slides were deparaffinized, rehydrated, and then boiled in antigen retrieval solution consisting of citrate buffer (pH 6.0) for the primary antibodies against Foxp3, CD4, CD8, CD68, CD20, and CD163. Sections were blocked using Antibody Diluent (Agilent Technologies) for 15 minutes, incubated with primary antibody for 30 minutes at 37°C then incubated with Opal Polymer HRP ELISA-labeled goat anti-mouse/rabbit IgG polymer (PV-8000) IgG for 10 minutes with the corresponding Opal 7-color fluorophores (Opal 620, 480, 570, 520, 650 and 780) for 10 minutes. Slides were restained using DAPI and sealed in ProlongGold (Thermo Fisher Scientific). After sections were stained, images were scanned in full using a TissueFAXS SL (7.1.120) panoramic tissue cell imaging system. Tissue and cell type identification and protein expression quantification of the scanned images were performed by StrataQuest (7.1.129) analysis software. The number of positive cells represented by each assay was determined based on the threshold value of that indicator, and the percentage of positive cells was further determined by calculating the number of positive cells as a percentage of the number of all cells in the section.

### Statistical methods

The experiment was designed according to the ‘3 + 3’ design principle. Continuous variables were expressed as mean and standard deviation, while categorical variables were expressed as number and percentage. The Kaplan-Meier method was used to describe outcomes from time to event, including progression-free survival and overall survival. Binomial proportions with 95% exact CIs were reported. adverse events were summarized using descriptive statistics. Non-parametric statistical tests were performed on lesion regression as well as biological samples to detect treatment-related changes over time. Group 1, Group 2 and Group 3 are used to denote the single irradiation dose 6Gy, 8Gy and 10Gy groups, respectively. Pre is used to indicate before treatment and Post is used to indicate after 2 cycles of immune checkpoint inhibitor treatment. P values less than 0.05 were considered statistically significant. All analyses were performed using SPSS Statistics 25.0 and GraphPad Prism software (version 8.0.2).

## Results

### Patient characteristics

Between August 2023 and May 2024, nine eligible patients participated in the study. The first three patients were assigned to the 6Gy group, the subsequent three to the 8Gy group, and the final three to the 10Gy group. The median age of the enrolled patients was 64 years (range: 42-71), and five of the patients were male. The baseline characteristics of the patients are shown in **Table 1**. The median follow-up duration was 8.3 months, with data cut-off on September 15, 2024.

**Table 1:** Patient characteristics.

Group	1			2			3		
dose SBRT level (Gy)	6			8			10		
Patient ID	1	2	3	5	6	7	8	9	10
Number of prior systemic therapies	4	4	4	4	5	3	4	5	4
Metastatic disease at diagnosis	Yes	Yes	Yes	Yes	No	No	No	No	No
Prior surgery	Yes	No	No	No	No	No	Yes	Yes	No
Prior radiotherapy	No	No	No	No	No	No	No	Yes	No
KRAS/BRAF mutation	Yes	Yes	No	Yes	Yes	Yes	No	NE	Yes
SBRT Median tumor size (cm)	4.6	6 2	6.8	4.1	6.2	6.2	4	6.5	6.2
SBRT Median tumor volume (cm3)	50.3	36.2	161.8	36.8	124.7	127.8	33.12	146.9	140.3
Number of high dose target areas	3	2	3	1	2	3	1	1	1
Number of low dose target areas	3	9	6	5	6	4	2	3	10
Number of radiotherapy sessions	4	3	4	3	4	4	3	5	3
Biological Equvalent Dose (Gy)	38.4	28.8	38.4	43.2	57.6	57.6	60	100	60
Zimberelima b treatment cycle	4	2	2	2	2	2	5	2	2
Efficacy results	PR	SD	PD	PD	SD	SD	SD	PD	PD
PFS (months)	2.9	1.6	1.6	1.6	2.2	1.6	3.6	1.4	1.6

CR, complete response; PR, partial response; SD, stable disease; PD, progressive disease; HPD, hyperprogressive disease. Responses according to RECIST 1.1.

### Safety and tolerability

**Figure 1:**
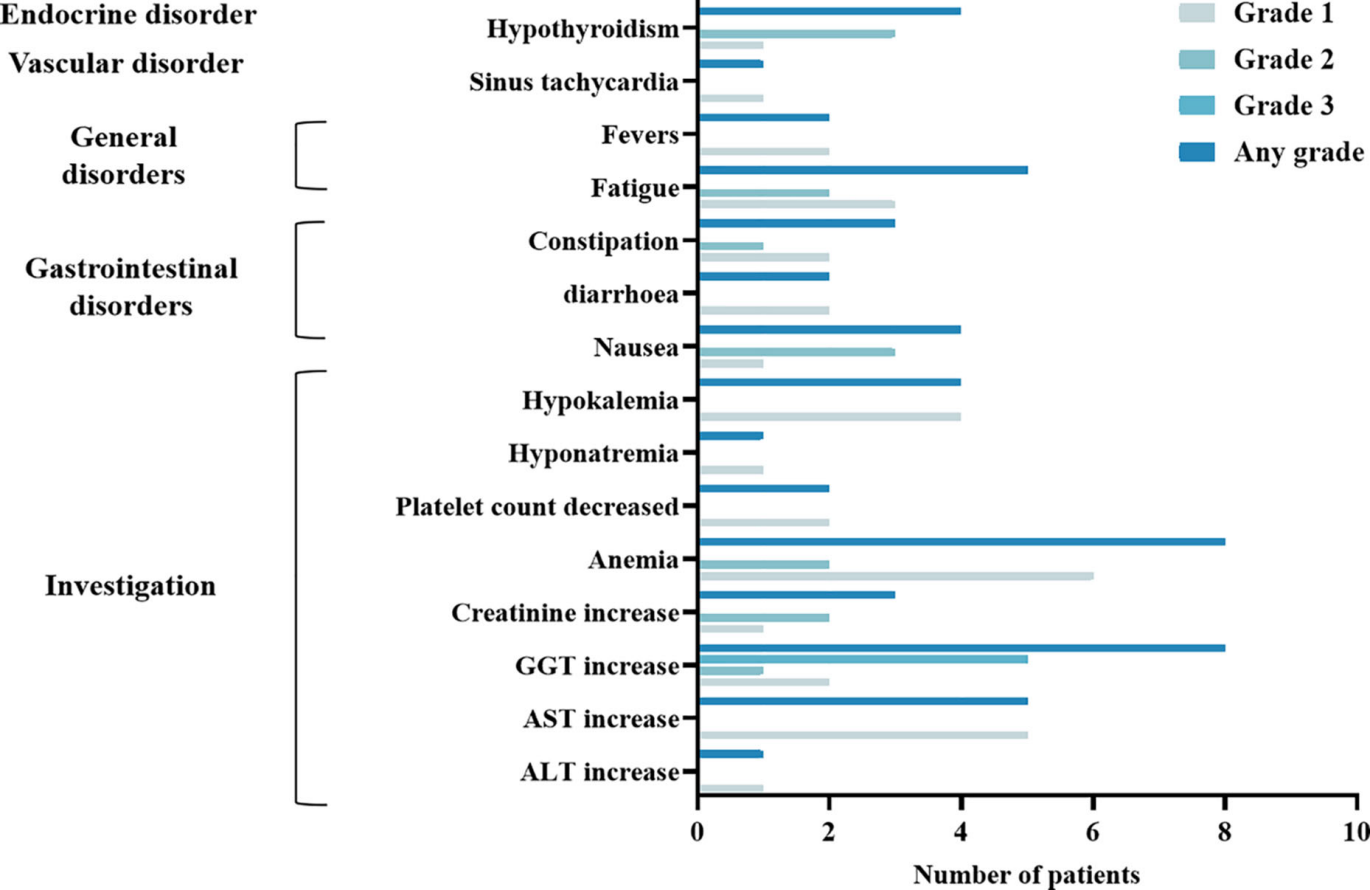
Overall trial safety. TRAEs (n = 9). System organ class and symptoms are shown. ALT Alanine-Aminotransferase; AST Aspartate-Aminotransferase; GGT Gamma-glutamyltransferase. The most common trAEs were Gamma-glutamyltransferase (GGT) increase (8 [88.9%]), Aspartate-Aminotransferase (AST) increase (8 [88.9%]), and anemia (8 [88.9%]).

The study did not cause any unexpected trAE, and was generally well-tolerated. All nine patients in the trial experienced at least one treatment-related adverse event (trAE). The most common trAEs were elevated γ-glutamyl transferase (GGT) (8 [88.9%]), elevated aspartate aminotransferase (AST) (8 [88.9%]), and anemia (8 [88.9%]). There were no significant differences in the severity and type of trAEs among the high-dose radiation therapy levels (*P*>0.05). An overview of general safety is provided in **Figure 1**.

### Antitumor activity and efficacy

**Figure 2:**
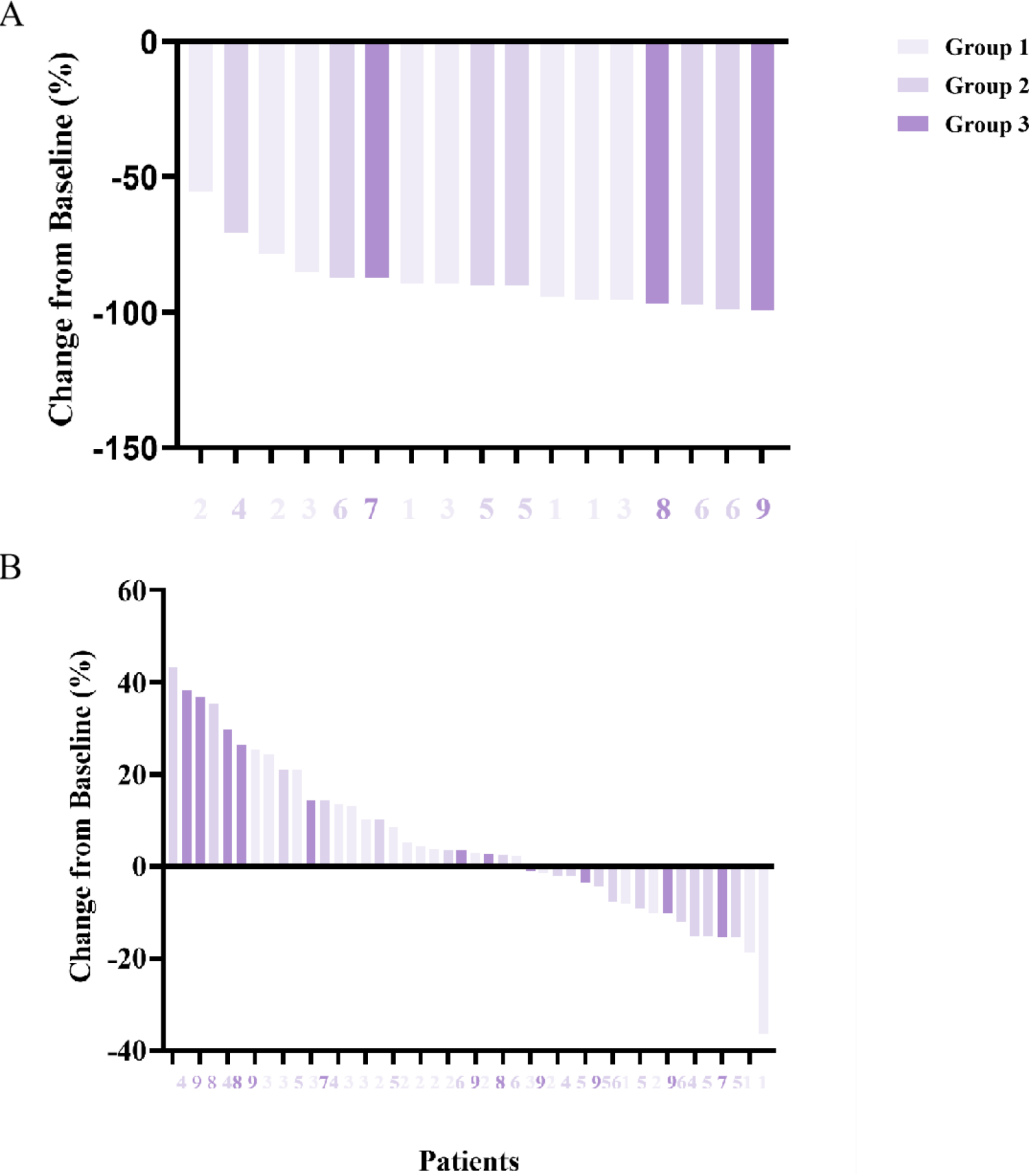
Radiological response was assessed for tumor regression. Recession of lesions in the high-dose area **(A)** and measurable lesions in the low-dose area **(B)** for each patient. Better regression of lesions in the high dose area than in the low dose area. (*P=0.0137, <0.05)*.

Among these 9 patients, 1 patient who received 6 Gy of irradiation achieved PR. The remaining 4 patients had SD and 4 patient had PD (**Table 1**). The ORR of the treatment was 11.11%. As shown in **Figure 2**, the high dose lesions regressed better than the low-dose areas, and the difference was statistically significant (*P*=0.0137, <0.05).

**Figure 3:**
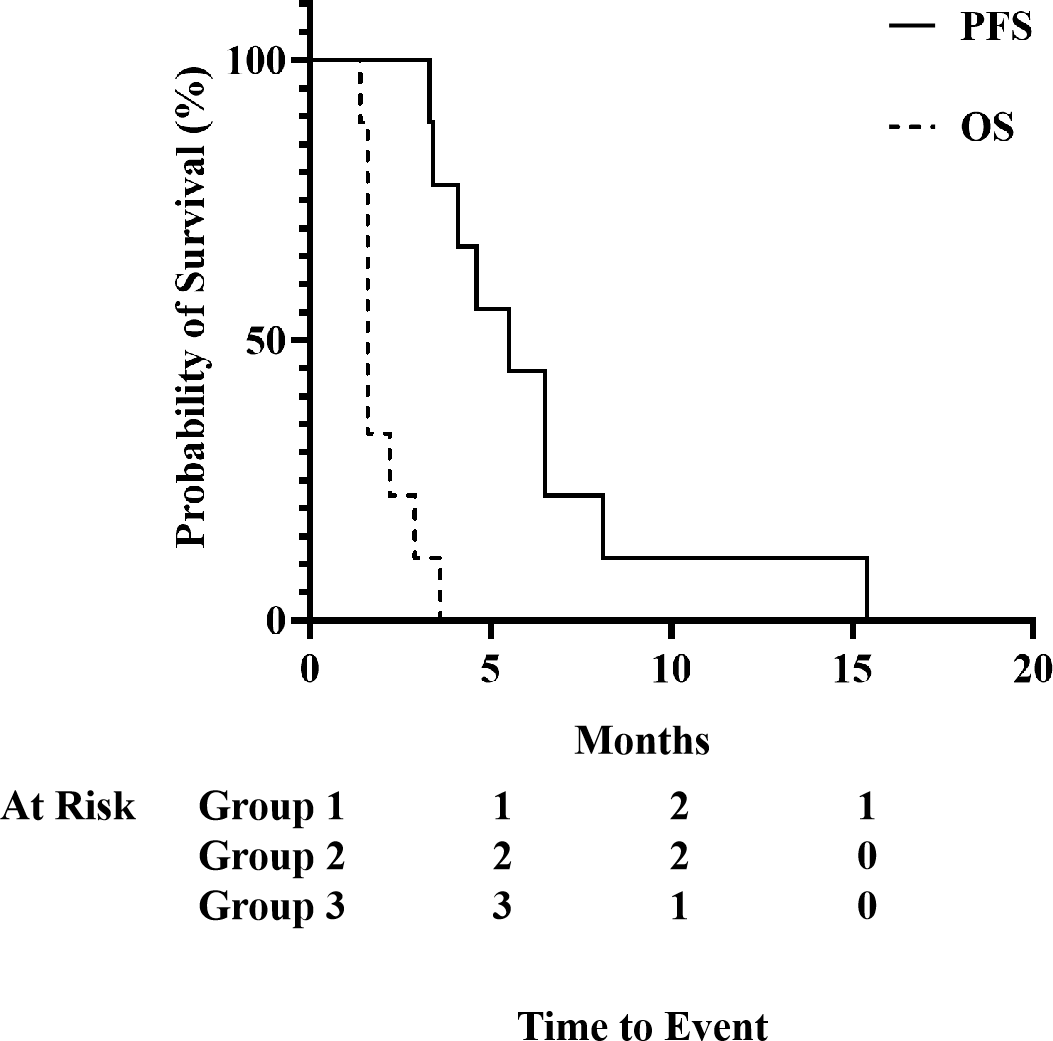
Kaplan-Meier survival analyses of progression-free survival (PFS) and overall survival (OS). After median a follow-up of 8.3 months, median PFS was 1.6 months (95% CI: 0.1 to 0.7), and median OS was 5.5 months (95% CI: 1.4 to 8.7).

As shown in **Figure 3**, the median progression-free survival was 1.6 months (95% CI: 0.1 to 0.7 months). The median overall survival was 5.5 months (95% CI: 1.4 to 8.7 months) with one patients living longer than 10 months (15.4 months). Tumor progression is the cause of death in all patients.

### Immunorelated analysis

**Figure 4:**
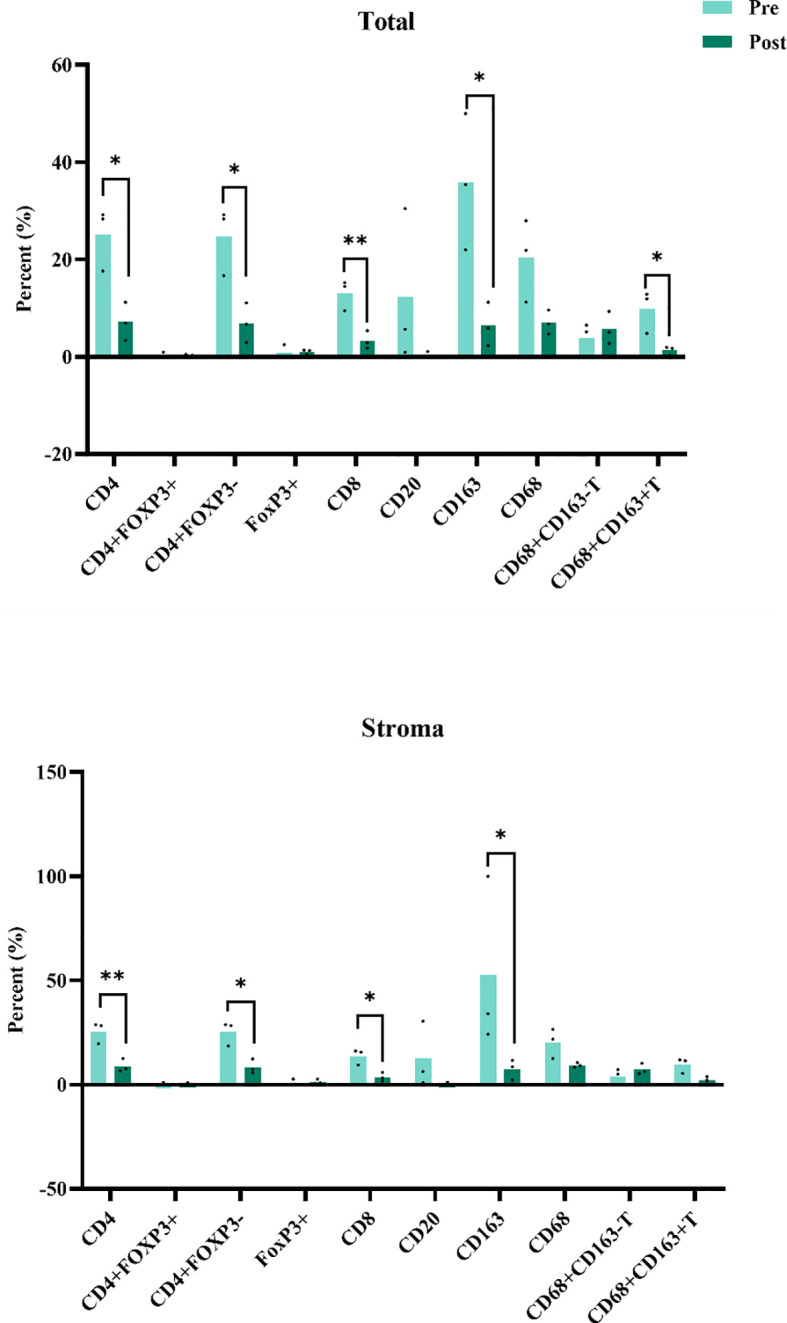
Multiple immunofluorescence results for paired samples from high-dose irradiated areas. Puncture samples were collected from the high-dose irradiated area before treatment(Pre) and after two cycles of zimberelimab injection (Post), respectively. Paired samples were subjected to multiple immunofluorescence assays to investigate different immune cell subpopulations. The percentage of CD4 T cells and CD8 T cells decreased before and after treatment, and the percentage of M2 macrophages decreased, whereas there were no significant changes in FoxP3+ and CD3 + FoxP3+ regulatory T cells (Tregs) as well as in B cells. *P<0.05, **P<0.01.

**Figure 5:**
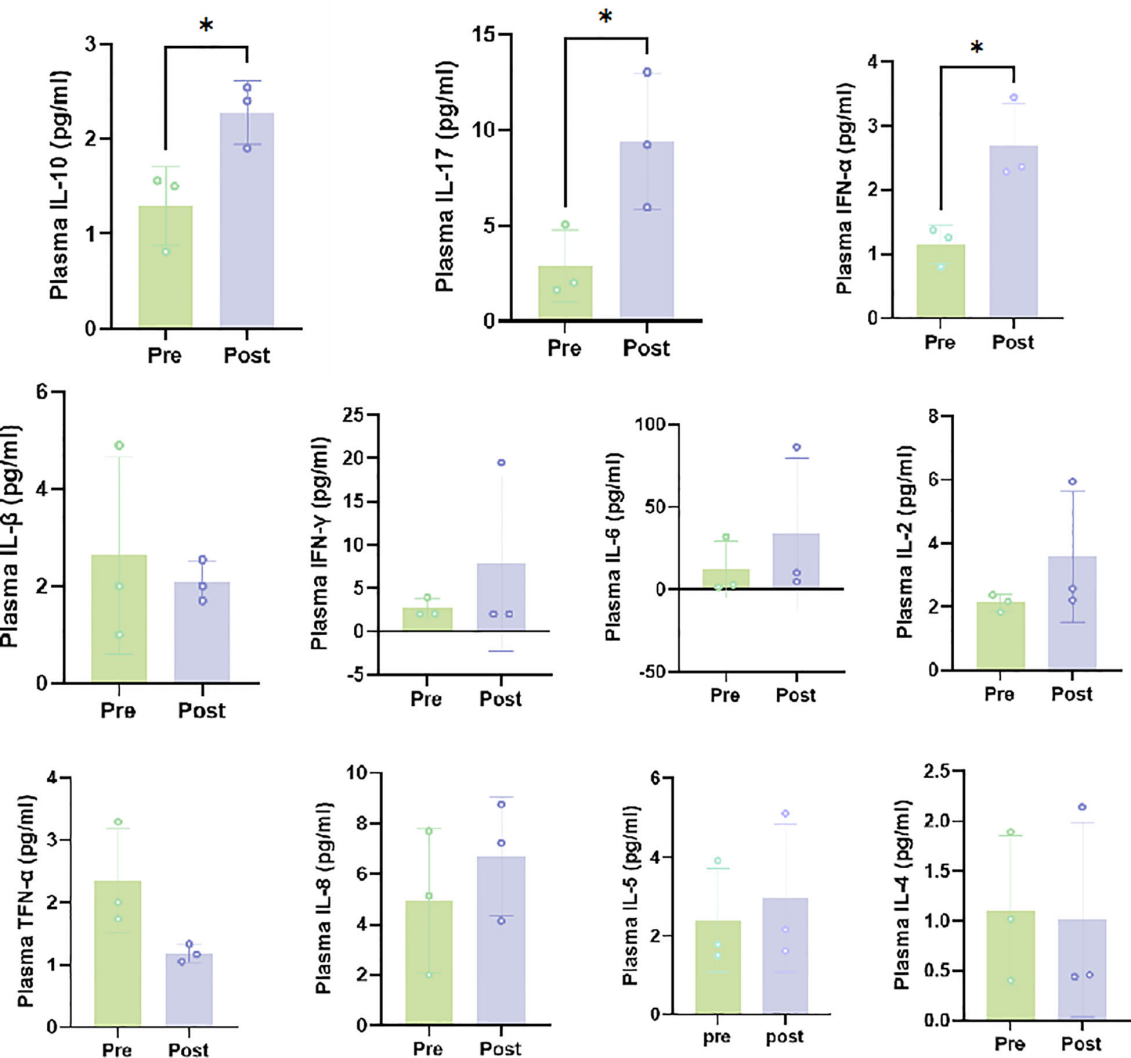
Plasma cytokines after high and low-dose radiation plus zimberelimab. Plasma samples were utilized to assess circulating cytokines. Eleven targets were identified, and plasma was tested with a Human Cytokine/Chemokine/Growth Factor ProcartaPlex Multiplex Immunoassay Panel **(A)** with Luminex xMAP technology. The above section describes treatment-related variations in cytokine levels. Treatment substantially elevated IL-10, IL-17, and INF-α levels. Results are presented as mean ± SEM. *P<0.05 indicates significant difference from baseline blood samples. Interferon (IFN), interleukin (IL), and tumor necrosis factor (TNF).

**Figure 6:**
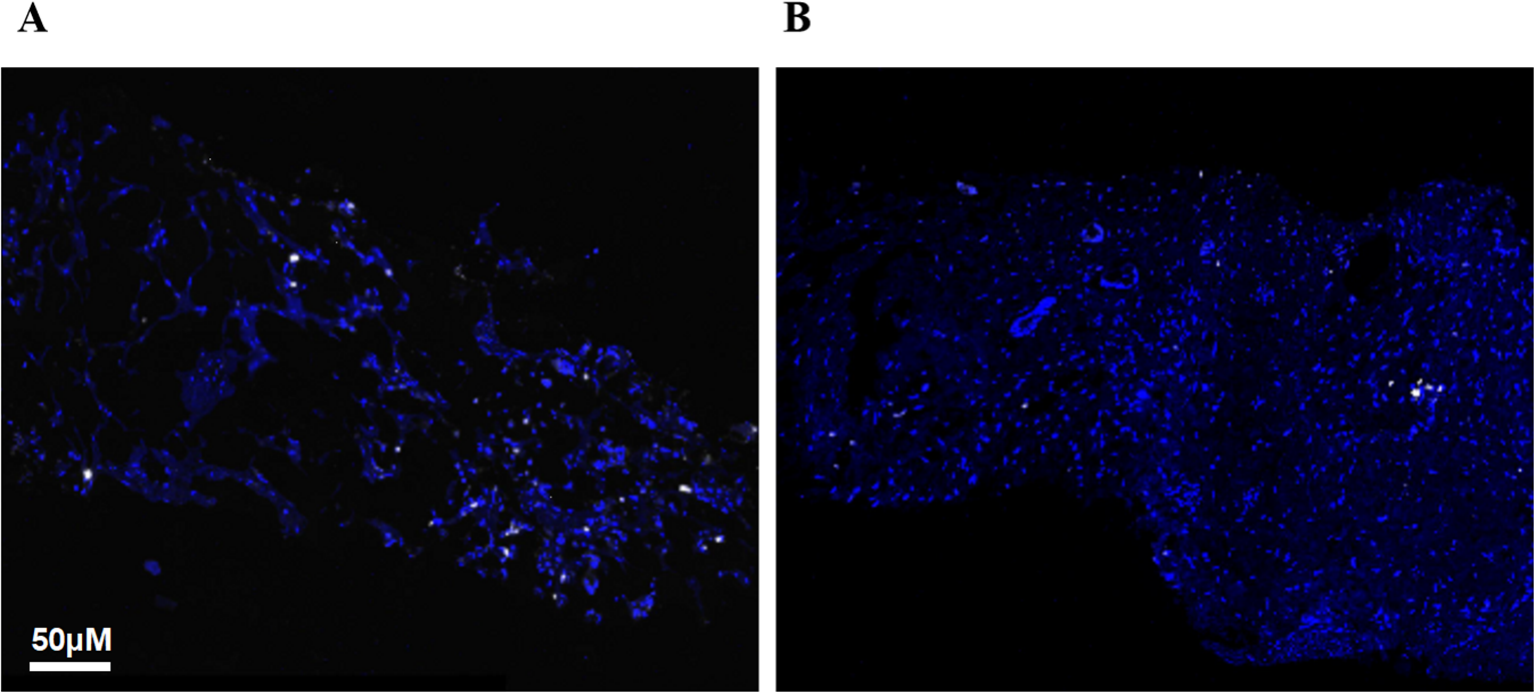
Immunofluorescence images of patient macrophages before and after treatment. **(A)** Pre-treatment; **(B)** CD163 antibodies are indicated in white, and M2 macrophages were reduced after treatment.

We compared the infiltration of T cells and macrophages in high dose irradiation areas before and after treatment in three patients. T cell types were identified using antibodies for CD4 (helper T cells), CD8 (cytotoxic T cells), and FoxP3 (regulatory T cells), while macrophages and granulocytes were identified using positive staining for CD68 (all macrophage types) and CD163 (M2 macrophages). Multiplex immunohistochemistry (mIHC) results indicated a decrease in the percentages of CD4 T cells and CD8 T cells and a reduction in M2 macrophages, while the proportions of FoxP3+ and CD3+ FoxP3+ regulatory T cells (Tregs) and B cells did not change significantly (**Figure 4**). Plasma circulating cytokine analysis showed increases in IL-10, IL-17, and INF-α following the triplet therapy. No significant differences were observed in cytokines such as IL-2, IL-5, IL-6, and IL-8 before and after treatment (**Figure 5**). Of note, one patient in group 1 achieved an overall survival of 15.4 months. This patient was initially diagnosed with rectal cancer with liver metastases and received neoadjuvant chemotherapy and surgical resection of the localized lesions. Recurrence of the liver lesions occurred 14 months after the end of treatment. In September 2023, high-dose radiotherapy (24Gy/4f) was started to three lesions in the liver, along with low-dose radiotherapy of 1.2Gy to the remaining three lesions. A total of 4 cycles of zimberelimab were administered 2 weeks after the end of radiotherapy. As shown in **Figure 6**, the patient’s tumor stroma showed a marked reduction in M2 macrophages after treatment.

## Discussion

Immunotherapy has shown significant efficacy in patients with mismatch repair deficiency or high microsatellite instability (dMMR/MSI-H) (15–18). However, its effectiveness in patients with MSS CRLM is limited. The median overall survival for patients with metastatic colorectal cancer undergoing third-line treatment is only 7-8 months (19, 20). It is now recognized that radiotherapy can activate immune responses. However, high dose radiotherapy alone may not be sufficient to overcome tumor-induced immunosuppression. Combining high dose radiotherapy with low-dose radiotherapy appears to overcome these limitations.

We designed a Phase I study of high low dose radiotherapy combined with ICIs in MSS CRLM patients. The results of the trial were not favorable, with only one patient achieving a PR. Nevertheless, our study was generally safe and tolerable and made two significant contributions: 1) One enrolled patient survived for 15.4 months. This suggests that this novel immunotherapy combination may influence the immunosuppressive environment in certain patients. 2) Immunoanalysis of paired samples before and after treatment enriched our understanding of this combination therapy.

As the prognosis for MSS CRLM is poor, especially for the inoperable patients, the median survival is less than 1 year. The study enrolled older patients who had received ≥3 lines of therapy, and poor patient status was associated with poor final OS data. However, there was still one patient who achieved an overall survival of 15.4 months, suggesting a survival benefit in certain populations from the combination treatment modality. The details of this patient have been described in detail in the results and hopefully will serve as a reminder for more prospective studies in the future.

Tumor-associated macrophages (TAMs) are a major component of TME (21). Based on their polarization state, TAMs can be classified into classically activated macrophages (M1) and alternatively activated macrophages (M2) (22). M2 macrophages have poor antigen-presenting abilities and secrete cytokines (including IL-10, TGF-β, CCL17, and CCL22) that induce an immunosuppressive TME. Therefore, high levels of M2 macrophages are generally associated with poor immunotherapy outcomes (23, 24). Different radiotherapy regimens and doses have complex effects on macrophages, leading to inconsistent results across studies (12). Some experiments suggest that high-dose radiotherapy induces the cGAS-STING pathway, promoting the polarization of macrophages toward the M1 phenotype (25). Conversely, *in vitro* studies by Felix et al. suggest that low-dose irradiation induces iNOS expression and increases NO secretion in macrophages (26). Our results showed a reduction in M2 macrophages after high-low dose radiotherapy, suggesting that this combination therapy promotes the reprogramming of TME toward an anti-tumor environment.

T cells are another crucial immune cell type in TME and are sensitive to radiation (27). CD4 T cells are generally considered more radioresistant than CD8 T cells, while Treg cells are the most radioresistant. Our multiplex immunofluorescence results showed a decrease in the percentages of CD4 and CD8 T cells after treatment, with no significant reduction in CD4 FOXP3- cells. Some studies have shown that the density of CD3 and CD8 lymphocytes in the tumor site correlates with disease-free and overall survival in rectal cancer patients receiving radiochemotherapy (28). Therefore, we suspect that the decrease in CD4 and CD8 T cells may be related to the unsatisfactory treatment outcomes.

During the analysis of cytokines, several cytokines (IL-10, IL-17, and IFN-α) showed an upward trend. IFN-α is considered an anti-tumor cytokine that can directly promote the differentiation of monocytes, activate macrophages and NK cells. IFN-α can also enhance the expression of MHC class I molecules in most cells, thereby enhancing antigen presentation by dendritic cells (DCs) and improving the function of effector T cells. In our study, the levels of IFN-α increased after treatment, which may confirm that the combination therapy stimulated the anti-tumor immune response. IL-10 has a bidirectional regulatory effect on tumor immunity. On one hand, IL-10 can induce tumor immune escape, promote tumor progression and metastasis by inhibiting the antigen-presenting function of APCs, suppressing T cell activation and immune response, and producing inhibitory factors. On the other hand, IL-10 can inhibit inflammatory factors that promote tumor development, thereby suppressing tumor-related inflammation, and enhancing the body’s anti-tumor effects (29, 30). This increases the cytotoxic activity of NK cells and CD8+ T cells, thereby enhancing the body’s tumor-killing effect and inhibiting tumor development and metastasis. In an IL-17-deficient mouse model, it was shown that IL-17-deficient mice are more susceptible to lung cancer and melanoma, which is direct evidence of IL-17’s protective role in anti-tumor responses (31). However, IL-17 promotes tumor growth by maintaining an inhibitory inflammatory environment through tumor angiogenesis, and by activating the STAT3 and NF-κB pathways to promote the expression of anti-apoptotic genes (32). Thus, the role of IL-17 for tumor therapy is also complex. After high- and low-dose radiotherapy combined with immune checkpoint inhibitors, the levels of IL-10 and IL-17 in the peripheral blood of patients increased. Although it cannot be concluded that the elevated levels of IL-10 and IL-17 are related to the patient’s treatment outcome, which was ultimately poor, it can be confirmed that this combined therapy stimulated the immune response through certain pathways.

In non-small cell lung cancer, Yin et al. demonstrated that delivering high-dose radiotherapy to the primary tumor, alongside low-dose radiotherapy to distant tumors and PD-1 blockade, significantly enhanced abscopal effects. This combination led to improved control of distant tumors (33).

Given the promising therapeutic effects of this triple therapy in other tumors and its immune response in microsatellite stable colorectal liver metastases (MSS CRLM), this combination holds substantial research value and significance.

The PACIFIC study conducted a retrospective exploratory analysis on the correlation between the initiation time of durvalumab after chemoradiotherapy and its efficacy. The results indicated that starting durvalumab within 14 days after completing chemoradiotherapy showed more pronounced benefits in progression-free survival (PFS) and overall survival (OS) compared to patients who started treatment 14 days or later. However, unlike the PACIFIC study, the LUN14-179 study required the initiation of pembrolizumab 4 to 8 weeks after chemoradiotherapy and found that patients who began treatment at 6 to 8 weeks demonstrated an improving trend in PFS and OS compared to those who started at 4 to 6 weeks. This suggests that our approach could be further studied and optimized regarding the timing of immune checkpoint inhibitor intervention and the sequence of chemoradiotherapy.

Stereotactic fractional radiotherapy (SFRT) is a mode of external beam treatment characterized by highly non-uniform dose distribution, and due to its immunomodulatory effects, it has gained significant attention in recent years. SFRT can deliver both low and high doses of radiation to the same tumor, thereby killing tumor cells while simultaneously activating the secretion and release of intra-tumoral cytokines. This promotes systemic inflammation and cytokine secretion, exerting anti-tumor effects on other non-irradiated tumors throughout the body. The combination of this technique with immune checkpoint inhibitors may provide significant advantages in advanced tumors (34, 35). Additionally, many targeted anti-angiogenic therapies can improve the structure and function of blood vessels, modulate the tumor microenvironment, and facilitate tissue perfusion as well as immune cell infiltration. The integration of targeted therapy with radiotherapy and immune checkpoint inhibitors is currently a hot research topic (36, 37).

We believe that future studies need to be more intensively investigated, so that the combination therapy can completely break the liver’s immune tolerance and exert even better efficacy in MSS CRLM. In the future, we will utilize single-cell sequencing and other techniques to observe changes in the immune microenvironment at the genetic level, aiming to gain a deeper and more comprehensive understanding of the effects of this triple therapy.

An important limitation of this study is the small sample size (n = 9) and the fact that our study did not determine the Recommended Phase 2 Dose(RP2D)and maximum tolerated dose (MTD). Despite this, the study provides unique insights into the immune mechanisms induced by this novel immunotherapy combination, adding new content to the sparse literature on the immune mechanisms of high-low dose radiotherapy.

## Conclusion

In summary, this triplet therapy showed good safety and tolerability in MSS colorectal cancer patients. High and low dose radiotherapy combined with ICIs demonstrated anti-tumor activity in the immune microenvironment of MSS CRLM. Future studies should consider adjusting the timing of immunotherapy combined with radiotherapy and determining the optimal radiation dose and frequency to completely overcome the immune tolerance of liver metastases.

## Data Availability

The original contributions presented in the study are included in the article/**Supplementary Material**. Further inquiries can be directed to the corresponding authors.
