# Supplementary material for: High and low dose radiotherapy combined with ICIs for MSS colorectal cancer patients with liver metastases: a phase I study (HaRyPOT)
Source: Front Oncol. 2025 Feb 6;15:1503517. doi: 10.3389/fonc.2025.1503517 (PMC11839429; doi:10.3389/fonc.2025.1503517)
Supplement: Supplementary file 1 [file DataSheet1.docx]

*Supplemental Figure 1: Full Eligibility Criteria*

Inclusion Criteria

- Patients must have histopathological confirmation of colorectal cancer by the Laboratory of Pathology of the NCI
- Patients must have radiologically confirmed liver metastasis.
- Patients must have progressed on > 2 lines of standard of care chemotherapy for colorectal cancer.
- Patient’s tumors must be documented to be microsatellite stable (MSS).
- Patients can have up to 10 non-central system lesions outside the liver.
- Patients must have at least one metastatic disease focus other than the irradiated field and be able to have a biopsy before and during treatment.
- Patients must have measurable disease by RECIST v 1.1 criteria.
- Age > 18 years.
- ECOG performance status 0-1
- Adequate hematological function defined by:

• white blood cell (WBC) count 23 x 109/L

• absolute neutrophil count (ANC) 2 1.5 x109/L

• lymphocyte count 2 0.5 x 109/L

• platelet count 2 100 x 109/L

• Hgb 29 g/ dL (more than 48 hours post-completion of blood transfusion))

• PT and PTT (seconds) < 1.2 x ULN. Patients who are anticoagulated do not need to meet criteria for PT and PTT

• INR < 1.2 x ULN. Patients who are anticoagulated do not need to meet criteria for INR.

•Adequate hepatic function defined by:

• a total bilirubin level :S 1.5 x ULN,

• an AST level : 5 x ULN in the presence of hepatic metastases,

• an ALT level : 5 x ULN in the presence of hepatic metastases.

- Adequate renal function defined by:

•Creatinine OR measured/calculated creatinine clearance (CrCl) <1.5 institution upper limit of normal OR 50ml/min/1.73m2 for participant with creatinine levels 1.5x institutional ULN.

- Patient must be able to understand and willing to sign a written informed consent document.

Exclusion Criteria

- Pregnant or breastfeeding women.
- The patients with portal vein thrombosis were excluded from this study.
- Patients currently on a corticosteroid dose greater than physiologic replacement dosing defined as 10 mg of cortisone per day or its equivalent.
- Patients with known brain metastases because of their poor prognosis and because they often develop progressive neurologic dysfunction that would confound the evaluation of neurologic and other adverse events.
- Patients with signs of liver failure, e.g. clinically significant ascites, encephalopathy, or variceal bleeding within 6 months prior to enrollment.
- Patients with active autoimmune disease or history of autoimmune disease that might recur, which may affect vital organ function or require immune suppressive treatment including systemic corticosteroids.
- History of idiopathic pulmonary fibrosis (including bronchiolitis obliterans with organizing pneumonia) or evidence of active pneumonitis on screening chest CT scan.
- Uncontrolled intercurrent illness including, but not limited to, ongoing or active infection, symptomatic congestive heart failure, unstable angina pectoris, cardiac arrhythmia, or psychiatric illness/social situations (within timeframes identified in the bullets below) that would limit compliance with study requirements.
- History of severe or unstable cerebrovascular disease.
- Pulse oximetry < 92% on room air
- Myocardial infarction within 6 months prior to enrollment
- History of myocarditis
- Sustained hypotension (<90/50 mmHg) or uncontrolled hypertension (>160/100 mmHg)
- Stroke within 6 months prior to enrollment.
- Patients with proliferative and/or vascular retinopathy.
- Significant vascular disorders (e.g. aortic aneurysm, requiring surgical repair or recent peripheral arterial thrombosis) within 6 months prior to enrollment.
- History of hemoptysis (> ½ teaspoon of bright red blood per episode) or active GI bleeding within 6 months prior to enrollment.
- Evidence of a bleeding diathesis or significant coagulopathy (in the absence of therapeutic anticoagulation).
- History of abdominal fistula or gastrointestinal perforation within 6 months prior to enrollment.
- HIV-positive patients are excluded because HIV causes complicated immune deficiency and study treatment can possess more risks for these patients.
- Prior autologous or allogenic hematopoietic stem cell transplant.
- Subjects with ascites.
- Patients with unhealed surgical wounds for more than 30 days.
- History of severe hypersensitivity reaction to any monoclonal antibody.
- Prior invasive malignancy (except non-melanomatous skin cancer) unless disease free for a minimum of 3 years prior to enrollment.


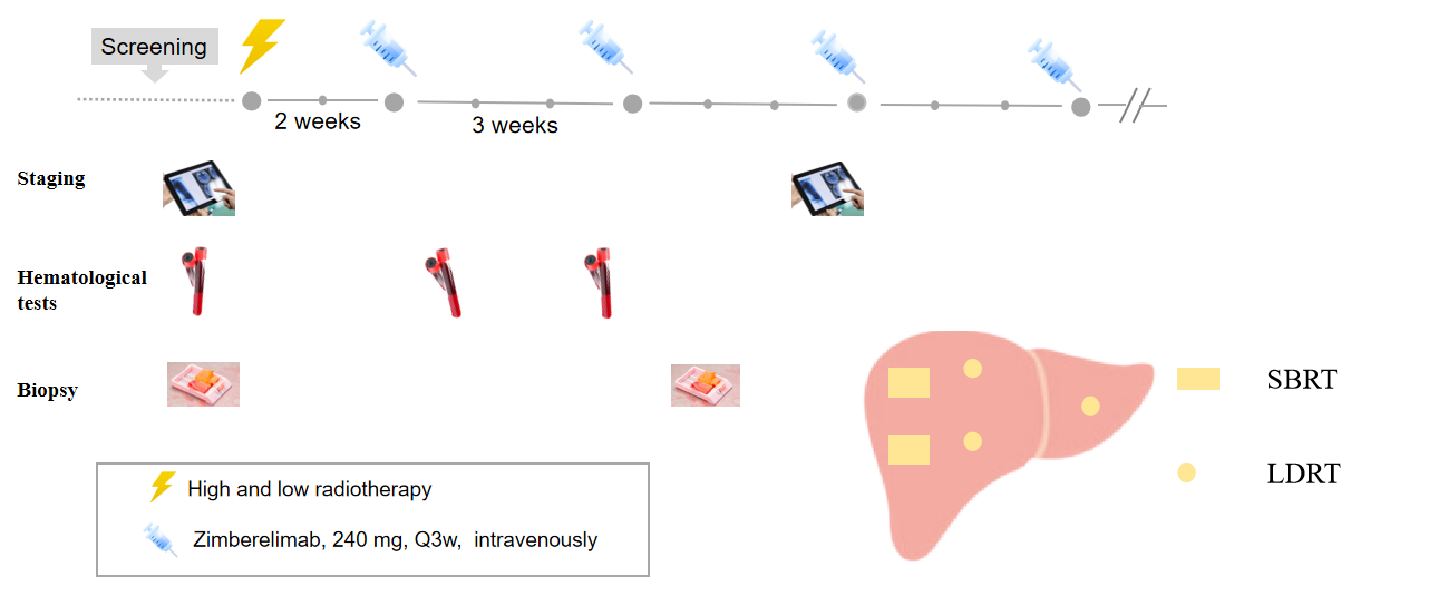


*Supplemental Figure 2. Study Schema*


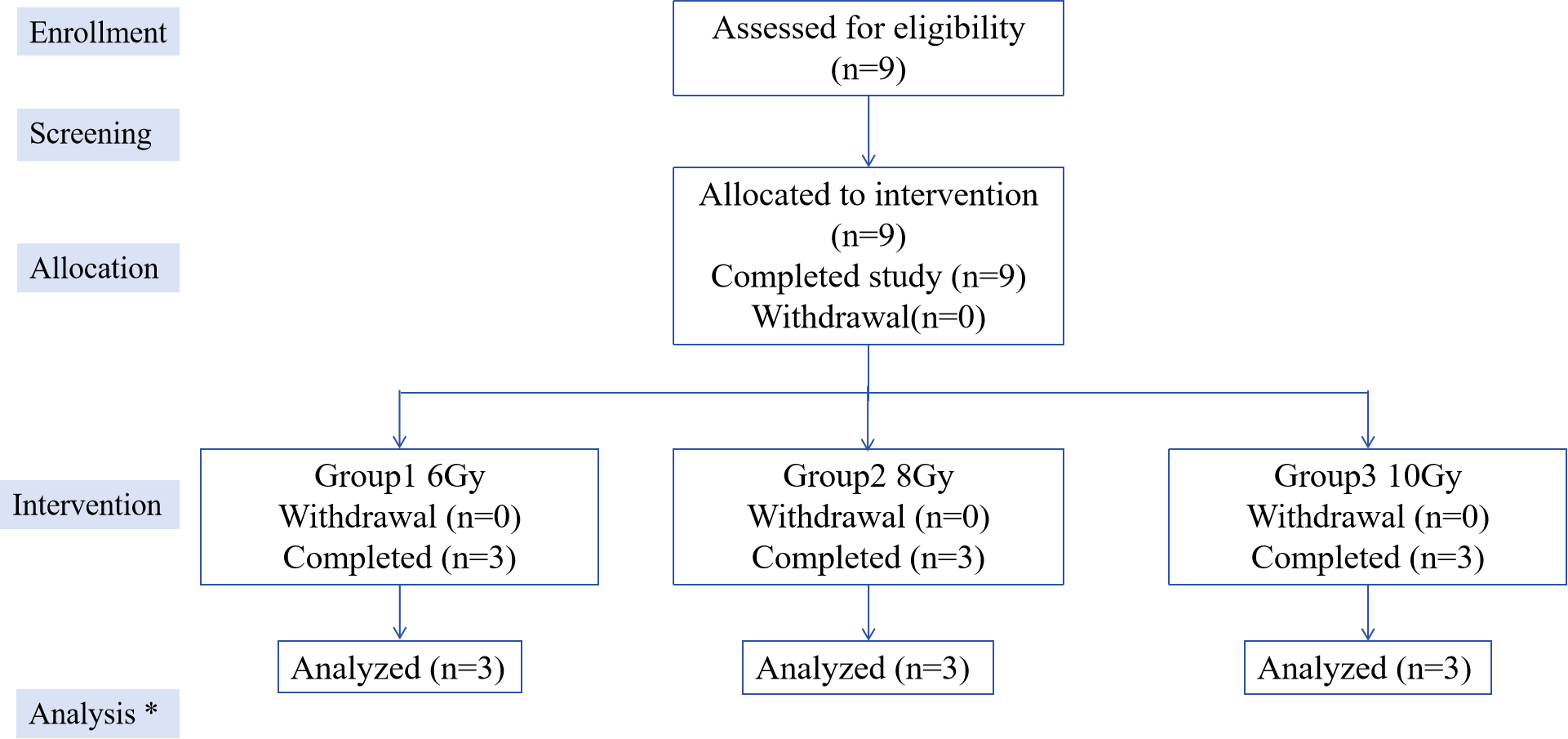


*Supplemental Figure 2. Study Disposition Consort Diagram*


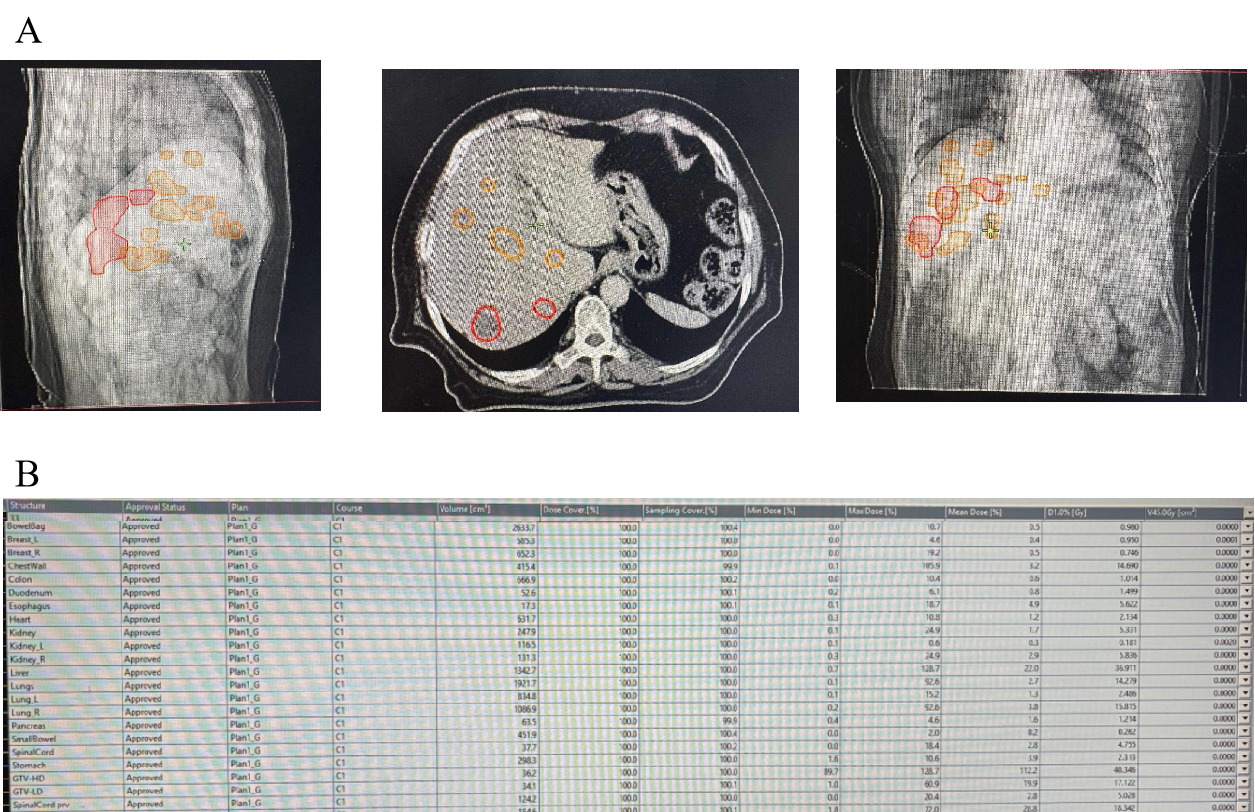


*Supplemental Figure 3. RT planning*

The Gross Target Volume of the liver lesion, with the high-dose radiation area in red and the low-dose irradiation area in orange (A), and the patient's plan evaluation (B).
